# First Report of *bla*_IMP-14_ on a Plasmid Harboring Multiple Drug Resistance Genes in Escherichia coli Sequence Type 131

**DOI:** 10.1128/AAC.00840-16

**Published:** 2016-07-22

**Authors:** Nicole Stoesser, Anna E. Sheppard, Gisele Peirano, Robert P. Sebra, Tarah Lynch, Luke W. Anson, Andrew Kasarskis, Mary R. Motyl, Derrick W. Crook, Johann D. Pitout

**Affiliations:** a Modernizing Medical Microbiology Consortium, Nuffield Department of Medicine, John Radcliffe Hospital, University of Oxford, Oxford, United Kingdom; b Division of Microbiology, Calgary Laboratory Services, Calgary, Alberta, Canada; c Department of Pathology and Laboratory Medicine, University of Calgary, Calgary, Alberta, Canada; d Icahn Institute and Department of Genetics and Genomic Sciences, Icahn School of Medicine, Mount Sinai, New York, New York, USA; e Clinical Microbiology, Merck and Co. Inc., Rahway, New Jersey, USA; f Department of Microbiology, Immunology, and Infectious Diseases, University of Calgary, Calgary, Alberta, Canada; g Snyder Institute for Chronic Diseases, University of Calgary, Calgary, Alberta, Canada; h Department of Medical Microbiology, University of Pretoria, Pretoria, South Africa

## Abstract

The *bla*_IMP-14_ carbapenem resistance gene has largely previously been observed in Pseudomonas aeruginosa and Acinetobacter spp. As part of global surveillance and sequencing of carbapenem-resistant Escherichia coli, we identified a sequence type 131 strain harboring *bla*_IMP-14_ within a class 1 integron, itself nested within an ∼54-kb multidrug resistance region on an epidemic IncA/C_2_ plasmid. The emergence of *bla*_IMP-14_ in this context in the ST131 lineage is of potential clinical concern.

## TEXT

The emergence of carbapenemases in clinically prevalent Escherichia coli lineages such as sequence type 131 (ST131) is a major problem for the management of patients infected with these strains (1, 2). Globally, five major transmissible carbapenemase enzymes predominate, represented by the KPC, OXA-48-like, NDM, VIM, and IMP families (2, 3).

An IMP metallo-beta-lactamase enzyme (IMP-1) was first detected in Japan in Pseudomonas aeruginosa in the late 1980s (4); since then, 52 genetically diverse *bla*_IMP_ gene variants 738 to 747 bp in length have been identified (5). Most *bla*_IMP_ variants have been isolated from either Pseudomonas or Acinetobacter spp. and demonstrate a degree of geographic structuring (6); however, some, such as *bla*_IMP-4_ and *bla*_IMP-8_, have emerged successfully in members of the family Enterobacteriaceae and are distributed over wider geographic regions (6, 7). Associations of *bla*_IMP_ with E. coli ST131 have, to date, been restricted to *bla*_IMP-4_ and *bla*_IMP-8_ in Taiwan, China, and Australia (8–11). As part of the Merck Study for Monitoring Antimicrobial Resistance Trends (SMART) (1), we identified an IMP-14-producing ST131 E. coli isolate, Ecol_732, that was isolated in Bangkok, Thailand, in 2012 and was sequenced in order to ascertain the genetic structures associated with this IMP variant in ST131.

Ecoli_732 was obtained from the urine of a hospitalized elderly male with a lower urinary tract infection. The MICs of ampicillin-sulbactam, piperacillin-tazobactam, cefoxitin, ceftriaxone, ceftazidime, cefepime, ertapenem, imipenem, amikacin, and ciprofloxacin were determined with microdilution panels prepared at International Health Management Associates, Inc. (Schaumburg, IL, USA), in accordance with 2015 CLSI guidelines. It tested nonsusceptible (i.e., either intermediate or resistant) to the above-mentioned agents. The MICs of colistin and tigecycline (determined by E tests) were 0.12 and 1 mg/liter, respectively.

DNA (chromosomal plus plasmid) was extracted from pure overnight subcultures of the isolate for both PacBio (long-read) sequencing and Illumina MiSeq (short-read) sequencing with the Qiagen Genomic-tip 100/G kit and the QIAamp DNA minikit (catalogue numbers 10243 and 51304; Qiagen, Valencia, CA), respectively. Preliminary *de novo* assembly of PacBio reads with HGAP3 was performed; resulting contigs were annotated with Prokka (12) and then trimmed on the basis of sequence/annotation overlaps in Geneious (version 9.04) (13). One-hundred-fifty-base paired-end MiSeq reads for each of the isolates were trimmed with Trimmomatic (version 0.35) (14) and then mapped to the corresponding PacBio assemblies with BWA mem (version 0.7.9a-r786) (15). Read pileups were inspected to confirm the structural integrity of the contigs and correct any small errors in the assembled contigs. Unmapped MiSeq reads were assembled with A5MiSeq (16) in order to identify any small plasmids (<7 kb) that may have been filtered out during the size selection process implemented as part of PacBio library preparation. Additional annotation focused on resistance genes and insertion sequences was performed with reference to the ResFinder (17), PlasmidFinder (18), and ISFinder (19) databases.

The Ecol_732 genome consists of a 5,009,900-bp chromosome and six plasmids, five of which could be fully resolved. These included pEC732-IMP14 (186,826 bp, IncA/C_2_), pEC732_2 (129,154 bp, IncFII/FIA/FIB/col), pEC732_3 (82,588 bp, IncB/O/K/Z), pEC732_4 (4,072 bp, untyped), and pEC732_5 (1,549 bp, untyped). A partial sixth *mob* plasmid fragment was also present (4,204 bp, untyped). The *bla*_IMP-14_ sequence in pEC732-IMP14 differed from the reference AY553332 by a single synonymous substitution (A249C), resulting in the same amino acid sequence. It was located within a 3,791-bp class 1 integron, In687 [*intI1-bla*_IMP-14_-*aac*(*6*′)-*qacEdelta-sul1*]. This integron is almost identical (single nucleotide difference, A925G) to that in Achromobacter xylosoxidans strain R4, which was cultured from a urine sample, also in Thailand (GenBank accession number KJ406505).

**FIG 1:**
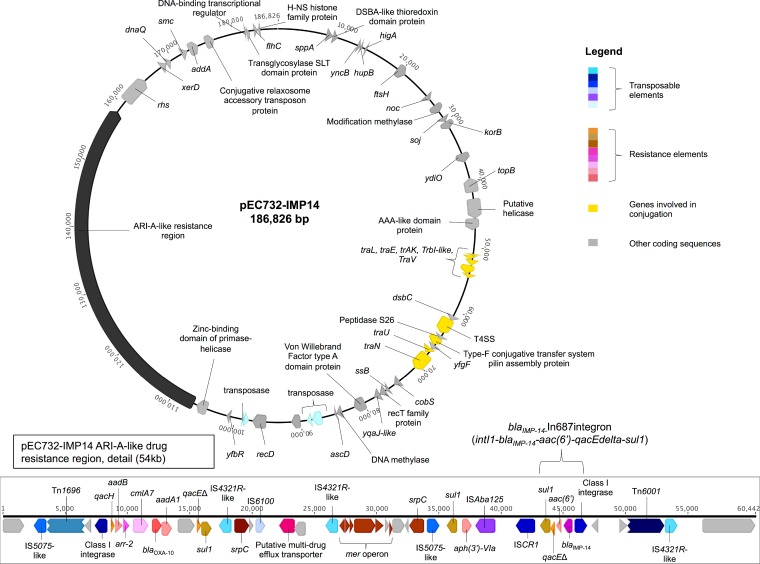
Plasmid pEC732-IMP14 with a detailed view of the ARI-A-like 54-kb resistance region (bottom).

The backbone of pEC732-IMP14 was highly similar to that of prototype type 1 IncA/C_2_ plasmid pRMH760 (RefSeq database accession no. NC_023898; from a Klebsiella pneumoniae strain) and other type 1 IncA/C_2_ plasmids (recently reviewed in reference 20), almost all of which also include a specific region designated ARI-A that contains a variable array of resistance genes and is located 1,711 bp upstream of *rhs* (20). Similarly, in pEC732-IMP14, the *bla*_IMP-14_-harboring integron was part of a much larger, 54,454-bp ARI-A-like region containing antimicrobial, heavy metal, and biocide resistance genes (Fig. 1), including those encoding resistance to beta-lactams (*bla*_OXA-10_, *bla*_IMP-14_), macrolides (drug efflux), rifampin (*arr-2*), sulfonamides (*sul1*), aminoglycosides [*aadA1*, *aadB*, *aph*(*3*′)-*IVa*, *aac*(*6*′)] chloramphenicol (*cmlA7*), chromate (*srpC*), mercury (*mer* operon), and quaternary ammonium compounds (*qac*). Some of these were part of a second, novel, integron designated In1286 (*intI1-qacH-aadB-arr-2-cmlA7-bla*_OXA-10_-*aadA1*).

**FIG 2:**
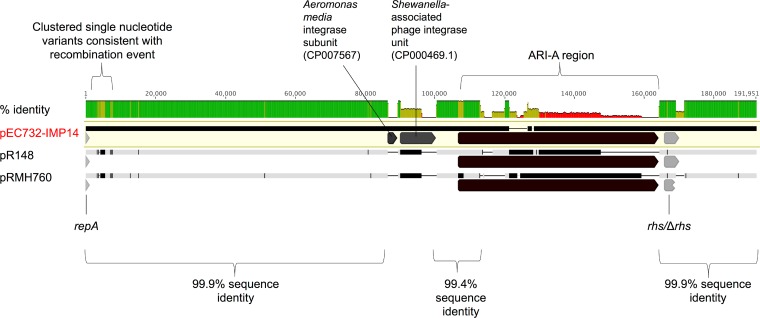
Alignment of pEC732-IMP14, pR148, and pRMH760 demonstrating mean percent pairwise identity over alignment columns (green, 100%; olive, 30 to 70%; red <30%; no color, 0%). Vertical black lines in the sequence bars represent differences between the nucleotide sequences with respect to the pEC732-IMP14 reference; thin horizontal black lines represent deleted regions. The *repA* gene, variable ARI-A resistance region, and *rhs* gene are indicated, as are the region of clustered single-nucleotide variants downstream of the *repA* region and two additional mobile genetic elements present in pEC732-IMP14 and absent from the other two sequences.

An alignment of pEC732-IMP14, prototype IncA/C_2_ type 1 plasmid pRMH760, and the only publicly available type 1 IncA/C_2_ sequence from Thailand, pR148 (RefSeq database accession no. NC_019380, from Aeromonas hydrophila [21]), demonstrates the genetic similarity of these plasmids (Fig. 2). All three sequences were >99% similar in the 1- to 86,573-bp region and in the ∼27.5-kb region downstream of ARI-A (Fig. 2). Differences in pEC732-IMP14 include a region of clustered single-nucleotide variants suggestive of a recombination event (region, 3,100 to 8,000 kb) and the acquisition of two integrase subunits (regions, 86,573 to 89,203 and 90,138 to 200,167 bp; Fig. 2). Interestingly, the pR148-containing A. hydrophila strain was identified on a Thai tilapia fish farm that had successively used several antimicrobial classes (21).

To date, *bla*_IMP-14_ has not been described in E. coli, to our knowledge, and has largely previously been reported in P. aeruginosa and Acinetobacter baumannii strains by several hospital centers in Thailand, in some cases as part of clonal outbreaks (22–25). Although *bla*_IMP-14_ is similarly associated with class 1 integrons in these cases, as in pEC732-IMP14, the wider plasmid contexts and sequences of these integrons in P. aeruginosa and A. baumannii strains have not been investigated. It is, however, conceivable that the *bla*_IMP-14_-harboring integron observed in pEC732-IMP14 and A. xylosoxidans strain R4 has been exchanged more widely with Pseudomonas and Acinetobacter spp. in Thailand. Class 1 integrons have been linked with the recent successful spread and expansion of another metallo-beta-lactamase, *bla*_VIM_, in IncA/C_2_ plasmids in members of the family Enterobacteriaceae in Greece (26) and *bla*_VIM_ and *bla*_IMP_ in Spain (27).

The presence of the extensively drug-resistant region observed here on an epidemic IncA/C_2_ plasmid in an E. coli ST131 strain from Thailand is therefore of concern and may represent wider, regional, horizontal dissemination of *bla*_IMP-14_ mediated by mobile genetic elements across bacterial families. The homology of pEC732-IMP14 with an A. hydrophila plasmid found on a fish farm and the presence of *bla*_IMP_-harboring plasmids in E. coli in other environmental (28) and animal sampling frames (29) suggest that the transmission network for IMP-positive E. coli may extend beyond the health care setting. Broad surveillance and control measures that are targeted at both community and health care contexts may be required to monitor and limit *bla*_IMP_ dissemination.

### Nucleotide sequence accession numbers.

Complete sequence data for Ecol_732 have been deposited in GenBank under BioProject number PRJNA316786. The accession numbers of the sequences are CP015138 (chromosome), CP015139 (pEC732-IMP14), CP015140 (pEC732_2), CP015141 (pEC732_3), CP015142 (pEC732_4), CP015143 (pEC732_5), and CP015144 (pEC732_6 [partial sequence only]).
